# Granulomatosis with polyangiitis (Wegener’s) as a necrotizing gingivitis mimic: a case report

**DOI:** 10.1186/1752-1947-8-297

**Published:** 2014-09-07

**Authors:** Katrina Genuis, Jesse Pewarchuk

**Affiliations:** 1 General Internal Medicine, South Island Division of GIM, Victoria General Hospital, 1 Hospital Way, Victoria, BC V8Z 6R5, Canada; 2 Island Medical Program, UBC Faculty of Medicine, Medical Science Building, University of Victoria, PO Box 1700 STN CSC, Victoria, BC V8W 2Y2, Canada

**Keywords:** Granulomatosis with polyangiitis, Wegener’s, Necrotizing gingivitis, ANCA, Rituximab

## Abstract

**Introduction:**

Granulomatosis with polyangiitis poses a significant diagnostic dilemma due its diverse presentations. Seemly isolated sites of disease, such as oral ulcers, may present to physicians working in primary care settings, the emergency room, and subspecialty fields as well as to dentists. Oral presentations are particularly challenging to identify and require a high index of suspicion and a detailed knowledge of the condition in order to diagnose and treat. We detail a case of granulomatosis with polyangiitis presenting as necrotizing gingivitis, one of the first of its kind to be reported.

**Case presentation:**

An otherwise healthy 32-year-old, Caucasian woman presented to various physicians with progressive, painful oral ulcers. Following consultations with multiple primary care physicians and subspecialties, an initial diagnosis of severe infectious necrotizing gingivitis was made resulting in combination antibiotic treatment as well as surgical debridement involving extraction of all maxillary and three mandibular teeth. With the discovery of a positive cytoplasmic anti-neutrophil cytoplasmic antibody and a constellation of associated systemic symptoms, our patient was subsequently diagnosed with granulomatosis with polyangiitis. The treatment regimen of rituximab and methylprednisone was chosen in consideration of our patient’s desire for future fertility and has been successful in inducing and maintaining remission.

**Conclusions:**

Following the case presentation, we review the current literature regarding granulomatosis with polyangiitis presentation, diagnosis and treatment. In discussing features of granulomatosis with polyangiitis presentation, diagnostic tests, and important new treatment options, we seek to enable physicians of all specialties to better recognize and begin appropriate treatment for this complex condition.

## Introduction

The identification of systemic vasculitides is one of most challenging diagnostic puzzles in clinical medicine. The diagnosis of the vasculitis granulomatosis with polyangiitis (GPA), previously Wegener’s, is particularly difficult due to its manifold presentations; from isolated dermatologic, to respiratory, to ophthalmologic manifestations, this dangerous systemic illness may be mistaken for an isolated complaint. We present the case of a 32-year-old woman who presented to several primary care physicians with the complaint of painful, progressing oral ulcers. The steps in reaching the surprising diagnosis of GPA are discussed as well as the choice of a specific treatment regimen that facilitated our patient’s desire for retaining fertility. We provide context for the case by reviewing the current literature with regard to various GPA presentations, criteria for diagnosis, and new treatment options.

## Case presentation

**Figure 1:**
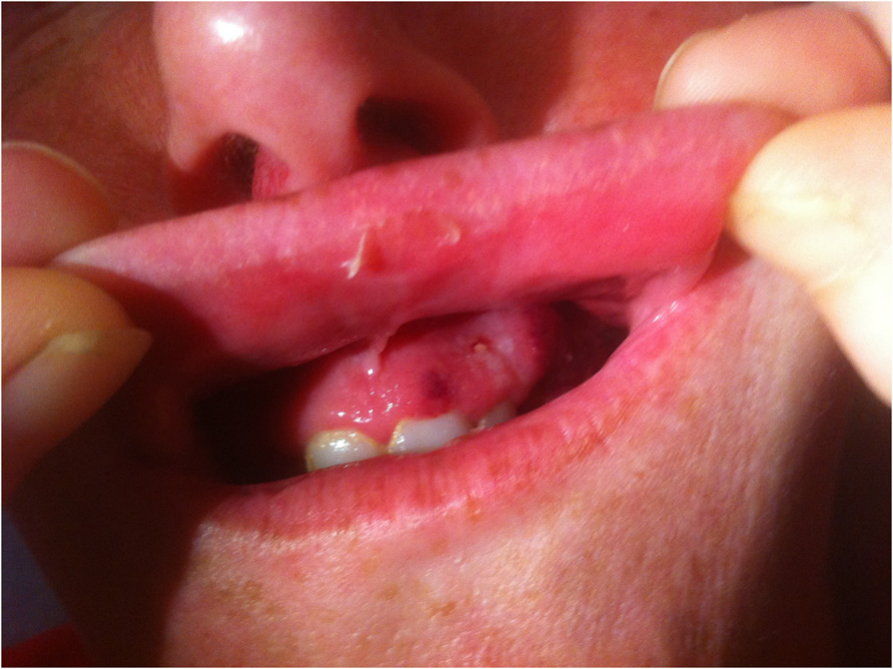
Initial appearance of ulcers on maxillary gingiva.

A 32-year-old, otherwise healthy Caucasian woman presented to her dentist with small, painful ulcers on her maxillary gingiva (Figure 1). An amoxicillin regimen for the presumed tooth infection was ineffective. Over the next month, the ulcer spread to involve the mandibular gingival, posterior alveolus, and retromolar trigone; concurrently; she became constitutionally unwell prompting a second course of antibiotics. After several days of worsening symptoms, our patient presented to the emergency department with unbearable trismus, necrotizing ulcers, and otalgia, requiring hospital admission. On admission, laboratory tests demonstrated an elevated white blood cell (WBC) count (16.9×10^9^/L), normal renal function (urea 7.2mmol/L, estimated glomerular filtration rate (eGFR) 116ml/min/1.73m^2^, creatinine 53umol/L), and a very high C-reactive protein (CRP) (193.3mg/L). Necrotizing gingivitis with osteomyelitis was diagnosed and metronidazole and ceftriaxone were prescribed under specialty care.

Within the first three days in hospital the necrotizing region expanded into the maxillary and mandibular bone and eroded through the maxillary antrum, resulting in aoroantral fistula. Extraction of all maxillary teeth and three mandibular teeth, as well as extensive surgical debridement and curettage of the maxillary bone were performed. Further progression prompted a second round of debridement and curettage five days later.

Our patient failed to recover with antibiotic treatment and surgery throughout these first nine days in hospital. Her severely declining clinical status was apparent; she experienced disabling fatigue, fevers, and drenching night sweats. Morning joint stiffness throughout the extremities as well as a numb patch on the right thumb was noted. In addition, our patient’s otalgia had worsened as she developed hearing loss associated with bilateral tympanic membrane perforation. Laboratory testing demonstrated leukocytosis, anemia (83g/L from 128g/L on admission), an increasing CRP (299.2mg/L from 193.3mg/L), and hematuria with red blood cell casts.

At this point, a positive cytoplasmic anti-neutrophil cytoplasmic antibody (c-ANCA) test and associated proteinase 3 ANCA (PR3-ANCA) levels (744.5U/ml) were received, suggesting a diagnosis of acute GPA. Our patient was reexamined in detail with specific attention to her history and physical examination features relevant to a GPA diagnosis. A thorough history revealed an active, healthy lifestyle without any significant stressors or past illnesses. Of possible consequence, we noted that she had occupational and personal exposure to various herbicides, black mold, and industrial solvents including acetone, without the use of personal protective equipment over the past 10 years. A physical examination revealed a gravely ill patient with trismus, hemorrhagic gingiva, inflamed otic canals obscured by serosanguinous discharge, bilaterally reduced air entry, inspiratory crackles as well as a patch of anesthesia on the palmar surface of the right thumb. Several 3 to 4mm erythematous nodular lesions on the dorsum of her left hand were observed.

Following the diagnosis of GPA, our patient was immediately treated with methylprednisone pulse therapy, and after several days intravenous (IV) cyclophosphamide was added. Relevant imaging tests and biopsies were carried out. Chest computed tomography (CT) imaging demonstrated multiple nodules, including a 2.8×3.0×1.9cm centrally cavitating pleural-based mass in the left apex, as well as bilateral pleural effusions. Histopathological examination of endobronchial biopsies from the right bronchus intermedius demonstrated necrotizing mucosal erosion and active inflammation.

Within days of treatment, dramatic improvement was seen. Our patient’s conductive hearing loss and otalgia began improving and laboratory test abnormalities including the previously elevated WBC count and CRP reversed. Due to our patient’s fertility concerns, rituximab - IV, 375mg/m^2^ - was substituted for cyclophosphamide. A four-week infusion cycle of rituximab along with prednisone, 60mg daily, was the core remission-inducing treatment regimen. A debriding procedure two weeks into treatment found that necrotic spread in her jaw had been halted. Chest CT imaging at one month demonstrated resolving lesions and no adverse changes.

Our patient was subsequently placed on a disease-modifying therapy regimen, including azathioprine and prednisone, and was followed by rheumatology, respirology and dental surgery specialists. Three months following her acute presentation, she underwent a partial surgical repair of the eroded maxillary antrum via iliac crest bone grafting. She continues to receive careful follow-up regarding her oral health post teeth extraction, repair of her maxillary antrum, as well as the severe damage done by the necrotizing vasculitic process to her left Eustachian tube and tympanic membrane. Our patient is regularly monitored through complete blood count (CBC), electrolyte, renal function, and CRP testing. Despite dealing with sequelae of the acute GPA, she has remained in remission from GPA for more than 15 months.

## Discussion

Until the latter 20^th^ century GPA was universally fatal, usually within weeks to months. Current diagnostic and treatment methods have reduced this mortality rate to less than 5 percent. Nevertheless, half of those diagnosed suffer from some form of long-term consequence due to residual damage from the condition or treatment [1]. It is critical that physicians recognize the condition early to minimize harm and reduce mortality risk in this potentially rapidly progressive disease.

### Presentations of GPA: critical recognition of clinical signs and symptoms

GPA can have dramatically varied presentations, ranging from an isolated ulcer or rash to fulminant multisystem crisis (Table 1). The underlying pathophysiological mechanism of tissue involves intravascular and extravascular granuloma formation associated with necrotizing vasculitis of small- and medium-sized arteries and veins. The frequency of this disease is equal between men and woman, and is more common in Caucasians. Although the average age of onset is 50, it can present at any age [1].

**Table 1:** Locations and forms of GPA presentation

**Site**	**Presentations**	**Frequency of disease occurrence at site throughout disease course (%)**
**Upper airway **[1]**,**[2]	Oral or nasal ulcers, strawberry gums, strawberry tongue, unresponsive upper respiratory tract or ear ‘infections’ , otitis, vestibular nerve injury, conductive or sensorineural deafness	92
**Lower airways **[1]**,**[2]	Subglottic stenosis, pulmonary nodular infiltrates, pulmonary hemorrhage ‘ground-glass’ infiltrations, pleurisy	85
**Kidneys **[1]**,**[3]	Rapidly progressing glomerulonephritis - necrotizing	80
**Musculoskeletal **[1,2]	Myalgias, migratory pauciarticular or polyarticular arthritis, arthralgias	67
**Eye **[1]**,**[2]**,**[4]	Scleritis, episcleritis, orbital pseudotumor, proptosis, dacrocystis	52
**Skin **[1]**,**[2]	Palpable purpura, ulcers, leucocytoclastic vasculitis, subcutaneous nodules, gangrene	46
**Nervous system **[1]**,**[2]	Peripheral neuropathy, mononeuritis multiplex, cranial neuropathy, meningitis	20
**Additional rare presentations **[2]**,**[3]	Myocarditis, pericarditis, cerebral vasculitis, mass lesions (brain, renal, lung, orbit, breast, prostate, ovary, parotid)	

Most patients experience prodromal constitutional symptoms, including fever, weight loss, night sweats, and joint stiffness. Gradual hearing loss and chronic nasal stuffiness or epistaxis are also common. The prodrome can last for months before progression occurs. Over 90 percent of patients first seek medical help for upper or lower airway symptoms, such as long-standing upper respiratory tract infections or shortness of breath [4].

The acute vasculitic phase can be global in scope, involving any system. Presentations of pulmonary hemorrhage or rapidly progressive glomerulonephritis are the most concerning; these can be fatal in hours or days [3]. Previously described oral manifestations of GPA include strawberry gingivitis, oral petechia and soft palate ulcers [5,6]. As described in the current case, patients transitioning from a prodromal phase to active vasculitis may present with features similar to a localized oral or cutaneous infection unresponsive to antibiotics. By maintaining a high index of suspicion and an awareness of the possible presentations, a physician is likely to perceive the clinical signs and symptoms of GPA and can move on to appropriate diagnostic testing: ANCA testing and histological examination.

### Diagnostic clues: distinguishing laboratory and histopathological tests for GPA

ANCA testing and/or histological examination of affected tissues represent the final steps necessary for a complete GPA diagnosis. As different vasculitides have differing ANCA test results, it is useful to carry out an ANCA test panel including two primary types of testing. Immunofluorescence testing determines c-ANCA or perinuclear anti-neutrophil cytoplasmic antibody (p-ANCA) presence. Enzyme immunoassays demonstrate the presence of PR3-ANCA and myeloperoxidase (MPO)-ANCA. c-ANCA, generally corresponding to PR3-ANCA positivity, has the greatest sensitivity (64 percent) and specificity (95 percent) for GPA, while p-ANCA and MPO-ANCA positivity generally corresponds to other vasculitides, such as microscopic polyangiitis and eosinophilic granulomatosis with polyangiitis (Churg-Strauss syndrome) [7]. In considering the use of ANCA testing it is important to note that the test sensitivity varies depending on disease progression. PR3-ANCA is most sensitive for GPA - sensitivity greater than 90 percent - in a setting with clear clinical signs and symptoms of GPA [8]. Additional laboratory tests that supported a GPA diagnosis include a high erythrocyte sedimentation rate (ESR), anemia, thrombocytosis and leukocytosis [3].

On histopathological examination, there are three classic features that can provide confirmation of a GPA diagnosis - granulomatous inflammation, aseptic vasculitis and necrosis [1,3,4]. These findings, however, are not necessary prior to proceeding with appropriate treatment, particularly when respiratory or renal damage is imminent. Moreover, obtaining a successful biopsy of affected tissue and demonstrating the triad of GPA features is notoriously difficult. Common biopsy sites include the upper respiratory tract or the lungs although even these sites do not have consistent biopsy success rates. As a result, a characteristic biopsy demonstrating GPA features is confirmatory, but not necessary.

### The changing landscape of GPA treatment

Although nonspecific intensive immunosuppressants have long been the successful mainstay of treatment for GPA, effective alternatives are on the horizon for patients with toxicity or unique immunosuppressant concerns. Current first-line treatment for inducing GPA remission consists of cyclophosphamide and glucocorticoids. This immunosuppressant regimen has saved thousands of lives, dropping the mortality rate from nearly 100 percent to 4 to 13 percent [1].

A randomized controlled trial with 149 ANCA-associated vasculitis patients demonstrated that cyclophosphamide can be effectively delivered in one of two forms: monthly IV treatments (15mg/kg) or daily oral treatments (2mg/kg/day). This cyclophosphamide dose was maintained until remission was achieved, up to a maximum of nine months [9]. While there is debate regarding the optimal glucocorticoid therapy regimen, patients with severe kidney or lung manifestations are typically treated with initial methylprednisone IV pulses for three days followed by oral prednisone, 1mg/kg/day. Those with less severe GPA forms may be treated with daily oral prednisone alone. Use of corticosteroids for greater than six months is not advised as it significantly increases patients’ infection risk [7].

While this therapy regimen has marked success, the significant morbidity associated with these medications cannot be ignored. Of particular concern is cyclophosphamide’s association with an increased risk of infertility, transitional cell carcinoma of the bladder, and myeloproliferative disorders [4]. Infection, the primary cause of death in the year following an initial GPA event, is also a significant risk.

With these side effects in mind, various alternative treatments are being actively researched and tested. The most promising alternative treatment to cyclophosphamide is rituximab, a chimeric B-cell monoclonal antibody. Rituximab has been explored as an effective choice for both inducing remission in ANCA-associated vasculitides. In July 2010, the *New England Journal of Medicine* reported Stone *et al.*’s multicenter, randomized, double-blind, double-dummy, noninferiority trial of rituximab against cyclophosphamide. This landmark trial successfully demonstrated noninferiority of rituximab to cyclophosphamide in inducing remission of ANCA-associated small-vessel vasculitis [10]. As such, rituximab (375mg/m^2^ for four weeks) is increasingly being used as substitute for GPA treatment. In the current case, the ability to provide rituximab as a fertility-protecting alternative made a significant impact in the patient’s comfort with the treatment and subsequent quality of life.

## Conclusions

Recognizing GPA is a true diagnostic challenge. Detailed history and physical examination are crucial to comprehensive understanding of presenting symptoms. Maintaining a degree of suspicion for vasculitides when a patient presents with constitutional symptoms or inflammation with antibiotic failure will allow for early recognition of this potentially devastating condition. Despite significant advances in GPA treatment, 50 percent of GPA patients experience some form of long-term disability due to the common rapid progression of the disease. In the current case, our patient may face long-term challenges associated with the loss of more than half of her teeth as well as left-sided hearing loss. Despite these unfortunate sequelae, effective recognition and treatment of this deadly condition prevented dialysis dependence, intensive care unit admission and possible death. Future advances in GPA diagnosis as well as increased awareness of the multiple possibilities of presentation will allow physicians to continue protecting the health of their patients through early GPA diagnosis and appropriate, prompt management.

## Consent

Written informed consent was obtained from the patient for publication of this case report and any accompanying image. A copy of the written consent is available for review by the Editor-in-Chief of this journal.

### Key points

GPA is a deceptive mimic of infection or malignancy, manifesting in numerous forms most often within the airway or the kidney.

In all branches of medicine, maintaining an index of suspicion for GPA allows physicians to make early diagnoses, which significantly reduces the morbidity/mortality of the condition.

Rituximab is an effective substitute for cyclophosphamide in acute GPA treatment regimens.

## Abbreviations

ANCA: anti-neutrophil cytoplasmic antibody; c-ANCA: cytoplasmic anti-neutrophil cytoplasmic antibody; CBC: complete blood count; CRP: C-reactive protein; CT: computed tomography; ESR: erythrocyte sedimentation rate; GFR: glomerular filtration rate; GPA: granulomatosis with polyangiitis; IV: intravenous; MPO-ANCA: myeloperoxidase anti-neutrophil cytoplasmic antibody; p-ANCA: perinuclear anti-neutrophil cytoplasmic antibody; PR3-ANCA: proteinase 3 anti-neutrophil cytoplasmic antibody; WBC: white blood cell.

## Competing interests

The authors declare that they have no competing interests.

## Authors’ contributions

KG interviewed the patient in the hospital setting, performed the literature review and drafted the case presentation and discussion. JP supervised the patient treatment in hospital and reviewed and edited the case report. Both authors read and approved the final manuscript.
